# PARSIMONIOUS MODELS OF LONG-TERM COGNITIVE DECLINE AND IMPAIRMENT PREDICTION IN MIDDLE-AGED ADULTS

**DOI:** 10.1093/geroni/igae098.1997

**Published:** 2024-12-31

**Authors:** Natascha Merten, A Alex Pinto, Adam Paulsen, Richard Chappell, Yanjun Chen, Corinne Engelmann, Laura Hancock, Carla Schubert

**Affiliations:** University of Wisconsin-Madison, Madison, Wisconsin, United States; University of Wisconsin Madison, Madison, Wisconsin, United States; University of Wisconsin Madison, Madison, Wisconsin, United States; University of Wisconsin Madison, Madison, Wisconsin, United States; University of Wisconsin Madison, Madison, Wisconsin, United States; University of Wisconsin Madison, Madison, Wisconsin, United States; Cleveland Clinic, Cleveland, Ohio, United States; University of Wisconsin Madison, Madison, Wisconsin, United States

## Abstract

Established risk scores for dementia prediction (e.g., the CAIDE and Framingham Risk Score) often focused on cardiovascular factors and have not been updated in more recent generations. We aimed to construct a parsimonious risk prediction model of 10-year cognitive decline and impairment using factors measured in midlife in a current cohort. This longitudinal study is based on N=1529(54% women, mean age 49years) Beaver Dam Offspring Study participants with data from baseline, 5-year and 10-year follow-up. We assessed hearing, vision, olfactory, motor, lifestyle, cardiovascular and general health factors, and blood-based neurodegenerative markers, and amyloid. We determined 10-year cognitive decline (trail-making test B time;10% most decline) and cognitive impairment (neurocognitive case review). We constructed least absolute shrinkage and selection operator logistic regression models with 10-fold cross-validation and evaluated predictive ability via receiver operating characteristic curves (AUCs). There were N=121 cognitive decline and N=217 cognitive impairment cases. The six top predictors for cognitive decline (age, income, motor function, olfactory function, peripheral artery disease, neurofilament light chain protein (NfL)) and for cognitive impairment (sex, motor function, olfactory function, self-rated vision, alcohol consumption, NfL) yielded AUCs of 0.80(95% confidence interval:[0.76-0.83]) and 0.73[0.69-0.77], respectively. In midlife, sensory and motor measures and NfL were among the best predictors of 10-year onset of cognitive decline and impairment. Only 6 factors were needed for acceptable to excellent AUCs. Other cohort studies should cross-validate these predictors. Risk scores for asymptomatic middle-aged people based on practical test batteries will be useful for population screenings to target early prevention and intervention strategies.

